# Cardiovascular, endocrine and behavioural responses to suckling and permanent separation in goats

**DOI:** 10.1186/1751-0147-52-51

**Published:** 2010-08-31

**Authors:** Louise Winblad von Walter, Lena Lidfors, Andrzej Madej, Kristina Dahlborn, Eva Hydbring-Sandberg

**Affiliations:** 1 Department of Anatomy, Physiology and Biochemistry, Faculty of Veterinary Medicine and Animal Science, Swedish University of Agricultural Sciences, SE-750 07 Uppsala, Sweden; 2 Department of Environment and Health, Faculty of Veterinary Medicine and Animal Science, Swedish University of Agricultural Sciences, SE- 532 23 Skara, Sweden

## Abstract

**Background:**

Suckling can be a peaceful or vulnerable event for goats and kids, whereas, separation is suggested as stressful. The aim of this study was to investigate physiology and behaviour in these two different situations in dairy goats.

**Methods:**

Four studies were performed with seven goats kept with their first-born kid in individual boxes. The goats were videotaped and heart rate and arterial blood pressure were recorded every minute by telemetry from parturition until 24 hours after separation. One to two days after parturition, Study 1 was performed with analyses of heart rate and blood pressure around a suckling. In Study 2, performed 3-5 days after parturition, blood sampling was done before, during and after suckling. Study 3 was performed 4-6 days post partum, with blood sampling before and after a permanent goat and kid separation. In addition, vocalisations were recorded after separation. Blood samples were obtained from a jugular vein catheter and analysed for plasma cortisol, β-endorphin, oxytocin, and vasopressin concentrations. Study 4 was performed during the first (N1) and second nights (N2) after parturition and the nights after Study 2 (N3) and 3 (N4). Heart rate, blood pressure and time spent lying down were recorded.

**Results:**

The kids suckled 2 ± 0.2 times per hour and each suckling bout lasted 43 ± 15 s. In Study 1, heart rate and blood pressure did not change significantly during undisturbed suckling. In Study 2, plasma cortisol (P ≤ 0.05 during suckling and P ≤ 0.01 five minutes after suckling) and β-endorphin (P ≤ 0.05) concentrations increased during suckling, but oxytocin and vasopressin concentrations did not change. In Study 3, the goats and kids vocalised intensively during the first 20 minutes after separation, but the physiological variables were not affected. In Study 4, heart rate and arterial blood pressure declined gradually after parturition and were lowest during N4 (P ≤ 0.05) when the goats spent longer time lying down than during earlier nights (P ≤ 0.01 during N1 and N3 and P ≤ 0.05 during N2).

**Conclusions:**

Suckling elevated plasma cortisol and β-endorphin concentrations in the goats. The intensive vocalisation in the goats after separation, earlier suggested to indicate stress, was not accompanied by cardiovascular or endocrine responses.

## Background

Suckling is usually considered a positive situation, whereas, abrupt and early separation of mother and young may have negative effects on the welfare of the animals [1]. The most common practice on dairy farms is to permanently separate mother and young immediately after parturition. After separation, the animals are often kept in the same facility. Swedish organic production states that mother and young separation should be performed after the colostrum period and not immediately post partum [2]. However, from a welfare point of view, it is questioned if separation after colostrum period may cause more distress than immediate separation. In cows, behavioural signs of arousal, *i.e *vocalisation, standing, sniffing, and placing the heads outside the pen, become more intense after separation if the cow and calf have spent longer time together [3]. In the present study, physiological and behavioural variables in goats were compared during suckling and in connection with separation after the colostrum period, which in goats last 3-4 days.

In goats, an exclusive bond between mother and young develops rapidly after birth, mainly due to olfactory cues from the kids [4-6]. A visual and acoustic bond is established within 4 hours after parturition [6], but soon after birth, goat kids hide and the goats either forage near their offspring separated from the herd or leave the kid unattended and follow the herd. In either case, goats can be classified as hider species the first days after parturition [7] and goats and kids only have contact for nursing [8]. When the kids join the herd, the mother and kid keep in contact through visual and acoustic signals [9]. If they are separated, vocalisation becomes intensive [10,11]. Vocalisation is considered a useful and reliable measure of acute distress in goats and kids [9,12,13] and in cattle [14,15,3]. Therefore, vocalisation was recorded in the present study.

In a stressful situation, the sympathetic nervous system and the hypothalamic-pituitary-adrenal-axis become activated. Heart rate and blood pressure increase when an activation of the sympathetic nervous system occurs. During the last 10 years, surgically implanted devices for continuous telemetric registrations of heart rate and blood pressure have been used by us, who may be the only group using this methodology in the goat. The method enables unique studies on the undisturbed, conscious goat. Therefore, changes in heart rate and blood pressure during suckling and separation were investigated, and the variables were recorded both during daytime and night time. During the nights, the effects of separation could be studied when the goats were undisturbed by daily management procedures. This was of interest since earlier results have shown that the heart rate gradually declines, but blood pressure does not change during the first week after parturition in goats where the goats and kids were permanently separated immediately post partum [16].

Change in plasma cortisol concentration is a variable commonly measured as an indicator of stress. In goats, cortisol is reported to increase i.e. at transportation [17-20] and in kids at separation [10,11], disbudding [13], and along with β-endorphin at disbudding and castration [21]. In sows, there is a correlation between cortisol and β-endorphin concentrations around weaning with higher levels before and on the day of weaning than the day after [22]. Therefore, changes in plasma cortisol and β-endorphin concentrations during suckling and separation were compared. As both oxytocin and vasopressin increase during suckling in goats [23], these hormones were also measured in this study.

The hypothesis of this study was that suckling is a peaceful event, whereas, sudden and permanent separation causes stress in goats with established bonding to their kids. The aim was to investigate the physiology and behaviour of dairy goats during both suckling and permanent separation of goats and kids.

## Methods

### Animals, housing and management

The study was conducted on an experimental herd at the Swedish University of Agricultural Sciences, Uppsala, Sweden. Seven female goats of the Swedish domestic breed *(Capra hircus)*, 2-3 years old, were studied. The goats were routinely kept together in a large pen with straw and wood shavings as bedding and were accustomed to handling. The goats were fed hay and concentrates at 7.30 h and 15.00 h and water and mineral stones were freely available. During night hours (18.00-06.00 h), the light was switched off and the goats were undisturbed by management routines. The goats gave birth on separate days, between 11.00 h and 20.30 h, from late February to early April. Six goats delivered two kids and one goat delivered one kid. When parturition was close, the goats were moved to individual boxes (1.2 × 1.5 m) in an adjacent room, in which all experiments were performed. The walls of the boxes consisted of a 30 cm solid edge with horizontal bars above so the animals could see out. In this herd, the goats usually deliver 1-3 kids. Therefore, to standardise the experiment, it was decided in advance that only the first-born kid would stay with its mother in the box. The second-born kid was moved before the goat had licked it, so only one mother-kid bond was established. None of the goats delivered three kids. The care of the animals and the experimental design were approved by the Animal Ethics Committee in Uppsala, Sweden.

### Physiological and behavioural recordings

Heart rate and diastolic, mean, and systolic arterial blood pressure values were recorded telemetrically every minute. The telemetric device was implanted in each goat at least three months before the study. As reported by Hydbring and colleagues [24], a transmitter body (Data Sciences Inc., St Paul, MN, USA) was placed subcutaneously on the lateral side of the goat's neck under general anaesthesia. The transmitter was connected to a fluid-filled catheter, which was tunnelled subcutaneously to the superficial temporal artery and further into the carotid artery. The blood pressure and heart rate signals were transmitted to a receiver placed above each box and digitised. The software package used was Data Quest IV (Data Sciences Inc., St Paul, MN, USA).

A catheter (Secalon, Viggo Products, Helsingborg, Sweden) was inserted under local anaesthesia (Xylocain^®^, Astra, Södertälje, Sweden) into one of the jugular veins of the goat when blood samples were taken. The catheter was inserted at least 30 minutes before the first blood sample to avoid the effects of insertion and was provided with an extension tube fixed to the neck of the goat with adhesive tape, so that it was possible to draw blood without restraining the animal. After insertion, and after each blood sample, the catheter was flushed with sodium citrate.

Behaviour was recorded by video cameras mounted above each box and connected to a time-lapse video recorder (Panasonic). The videotapes were analysed by the same person. Vocalisation of the goats was recorded with a Compact VHS video camera (JVC), and vocalisation by the kids was recorded by direct observation.

### Study 1, Heart rate and blood pressure during suckling in the undisturbed goat

**Figure 1:**
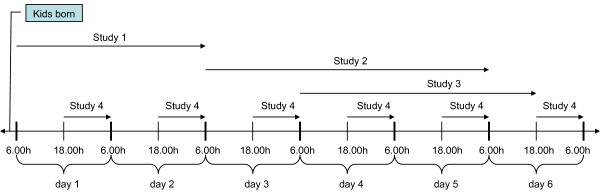
**Timeline of the study design (Study 1, Study 2, Study 3 and Study 4)**. The timeline shows six days (6.00 h-18.00 h) and nights (18.00 h- 06.00 h) after parturition. The horizontal arrows indicate during which days the four studies were performed.

The first study (Study 1) was performed 1-2 days after parturition (Figure 1). Heart rate and blood pressure were recorded telemetrically and the goats were continuously videotaped after parturition. The number of sucklings per hour and the average duration of each suckling were calculated from the video recordings. Suckling was defined as "kid standing with its head close to the mother's udder and the mother standing still". The video recordings were used to search for a suckling bout in the undisturbed goat, occurring between 24 hours and 48 hours after parturition. The suckling had to fulfil the criteria that it was a single suckling with no other suckling during 30 minutes before and after, and the suckling had to occur after 10.30 h to be comparable in time with suckling combined with blood sampling, performed in study 2. The cardiovascular data from the selected suckling was located for each goat and the mean values were calculated.

### Study 2, Heart rate, blood pressure and hormones during suckling

The second study (Study 2) was performed 3-5 days after parturition (Figure 1), and in addition to heart rate and blood pressure recordings, blood samples were taken from the goats. Control samples were taken at 10.00 h and 10.30 h and in between, the goats were left undisturbed. After the second sample, the goats and kids were closely watched. When the kid oriented itself towards the udder of its mother, the observers immediately, but cautiously, went into the box and when the kid suckled, the third blood sample was taken. Blood samples were then taken after 5, 15, and 30 minutes. After withdrawal of the last blood sample, the catheters were removed.

### Study 3, Heart rate, blood pressure, hormones, and behaviour at permanent separation

The third study (Study 3) was performed the day after Study 2, 4-6 days after parturition (Figure 1). Heart rate and blood pressure recordings were made and a first blood sample was taken from the goats at 10.00 h. The goat and kid were then left undisturbed until 10.25 h, when the second blood sample was withdrawn. At 10.30 h, the kid was removed to another room, so the goat and kid could hear but not see each other, and the third blood sample was taken. Further blood samples were taken 5, 10, 15, 30, 60 and 120 minutes after separation. After the last blood sample, the catheter was removed. The goats were milked in the late afternoon.

Vocalisation rate was defined as "every vocalisation with a pause between two occurrences". The vocalisation rate was counted during 120 minutes after separation, divided into twelve 10-minutes periods. For each 10-minutes period, the mean for both goats and kids was calculated. Lying down was defined as *"*the goat lying with its legs folded and its chest in contact with the floor". Latency time from separation until lying down was defined as "time from the moment the kid was separated from the mother until the goat laid down the first time".

### Study 4, Heart rate, blood pressure and time spent lying down at night

The fourth study (Study 4) was a comparison of the mean values of heart rate, blood pressure and total duration time spent lying down between the first and second nights after parturition, and the nights after Study 2 and Study 3 (Figure 1). The total duration spent lying down each night was recorded from the videotapes. The nights started at 18:00 h and ended the next morning at 06:00 h.

### Hormone analyses

Blood was drawn into ice-chilled tubes containing K_3_-EDTA and aprotinin (Trasylol^®^, 4 000 KIE/10 ml tube, Bayer, Leverkusen, Germany) and was centrifuged (4°C, 20 minutes, 1500 × *g*). The plasma was stored at -20° for 24 hours and thereafter at -70° until assayed. Analyses of plasma cortisol, β-endorphin, oxytocin, and vasopressin were validated for goat plasma and performed with radio-immuno-assay methods described by Hydbring et al. [25]. The intra-assay coefficient of variation was: ≤ 10% between 28-1380 nmol/l for cortisol; ≤ 10% between 7-120 pmol/l for β-endorphin; ≤ 10% between 21-744 pmol/l for oxytocin; and, ≤ 10% between 1.6-60 pmol/l for vasopressin. The least detectable value was 4.0 nmol/l for cortisol, 1.5 pmol/l for β-endorphin, 3.0 pmol/l for oxytocin, and 0.3 pmol/l for vasopressin.

### Statistics

Behavioural and hormonal data are presented as means ± standard error of the mean (S.E.M). Data were examined by repeated measurement ANOVA (mixed procedure) of the Statistical Analysis System (SAS Institute Inc. 2003). Hormonal differences within studies were tested through differences in least squares means against the first control sample. Differences in heart rate and blood pressure values within days were analysed with Bonferroni's Multiple Comparison Test. The coefficient of variation (CV) values for heart rate and mean blood pressure were compared during periods with and without blood sampling. The difference between respective CV values was tested by t-test. Differences in behavioural data were tested with the non-parametric Wilcoxon Signed Ranks test. Differences in heart rate, blood pressure and time spent lying down were tested during the nights (18.00 h-06.00 h) when goats were undisturbed. The level of significance was set at P ≤ 0.05.

## Results

### Study 1, Heart rate and blood pressure during suckling in the undisturbed goat

**Figure 2:**
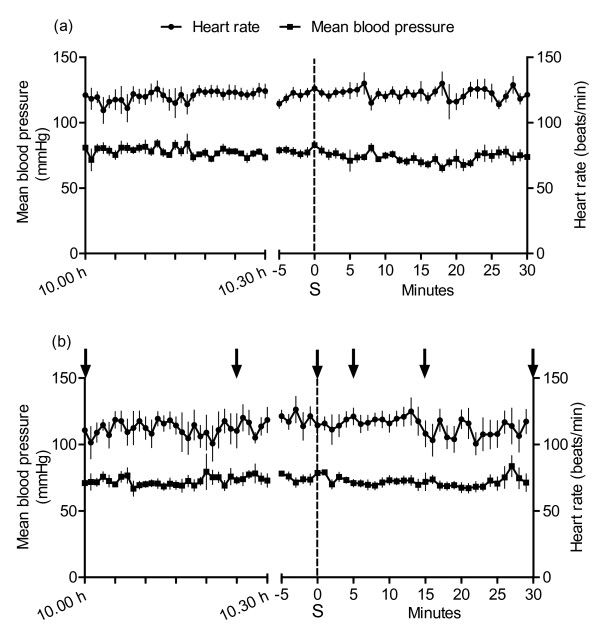
**Heart rate and blood pressure during suckling (Study 1 and Study 2)**. Means (± SEM) of heart rate and mean arterial blood pressure in seven goats before, during, and after undisturbed suckling (a) and before, during and after suckling with blood sampling (b). The recordings made at 10.00 h-10.30 h were taken on the same day as the first recorded suckling (S). Due to the different times for each goat, the curves to the right start at -5 minutes before the suckling (S). Arrows point at the time when blood samples were taken (Figure 3). Heart rate (P ≤ 0.001) and blood pressure (P ≤ 0.05) fluctuated more and the individual differences were higher on the day with blood sampling (b) than on the day with undisturbed suckling (a). No significant changes related to suckling were found.

During the first 24-48 hours post partum, the kids suckled on average 2 ± 0.2 times per hour and each suckling bout lasted approximately 43 ± 15 seconds. The average time until a suckling was observed after 10.30 h, without other suckling 30 minutes before and after, was 88 ± 18 minutes in the undisturbed goats. Both heart rate and blood pressure fluctuated during the day with individual differences, but there were no significant changes in heart rate or blood pressure related to suckling (Figure 2a).

### Study 2, Heart rate, blood pressure and hormones during suckling

**Figure 3:**
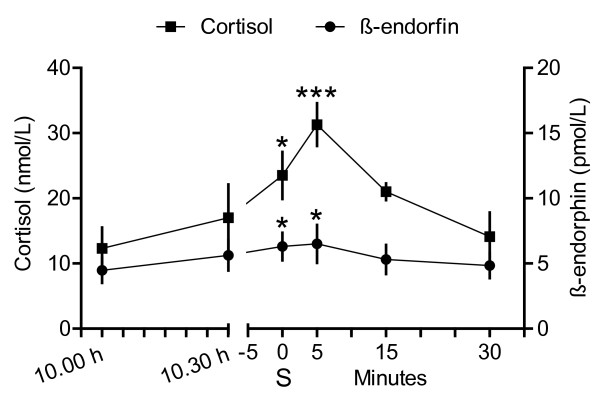
**Plasma concentrations of cortisol and β-endorphin during suckling (Study 2)**. Means (± SEM) of plasma concentrations of cortisol and β-endorphin before, during, and after suckling in seven goats. Blood samples were taken at 10.00 h and 10.30 h and on the same day as the suckling experiment, and then at suckling (S), and at 5, 15 and 30 minutes after suckling. * = P ≤ 0.05 and *** = P ≤ 0.001 indicates significant differences compared to the first sample (10.00 h).

It took 70 ± 10 minutes until a single suckling was observed. Heart rate (P ≤ 0.001) and blood pressure (P ≤ 0.05) fluctuated more and the individual differences were higher than in study 1, performed on the previous day (Figure 2). No change in blood pressure and heart rate was observed during suckling combined with simultaneous blood sampling (Figure 2b). Plasma concentrations of cortisol and β-endorphin were elevated during suckling (P ≤ 0.05) and the sample taken 5 minutes later (P ≤ 0.001 for cortisol and P ≤ 0.05 for β-endorphin), but after 15 and 30 minutes, the difference was not significant compared to control samples (Figure 3). The plasma oxytocin concentration was 21 ± 2 pmol/l and the vasopressin concentration was 1.0 ± 0.03 pmol/l in the first sample and did not change significantly during the experiment.

### Study 3, Heart rate, blood pressure, hormones and behaviour at permanent separation

**Figure 4:**
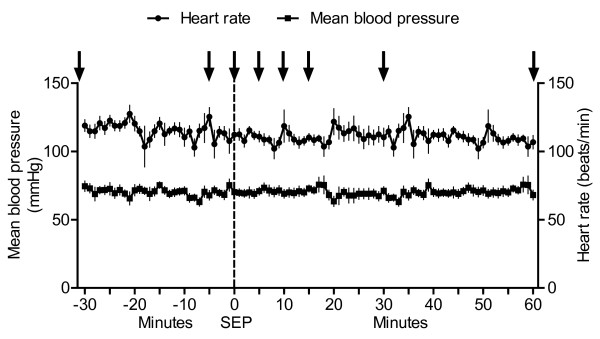
**Heart rate and blood pressure during separation (Study 3)**. Means (± SEM) of heart rate and mean arterial blood pressure in seven goats before, during, and after separation (SEP) of their kids. Arrows point at the time when blood samples were taken. No significant changes were found at separation.

**Figure 5:**
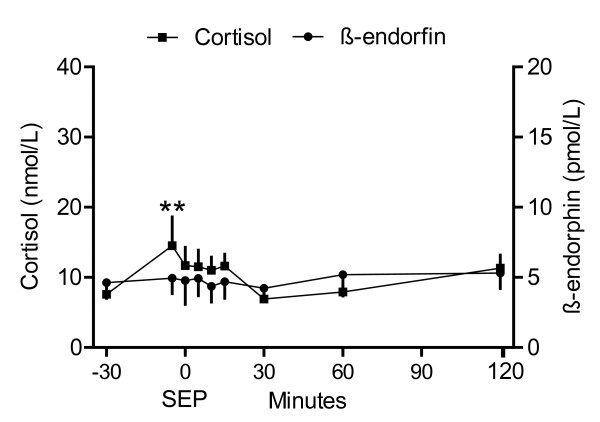
**Plasma concentrations of cortisol and β-endorphin during separation (Study 3)**. Means (± SEM) of plasma concentrations of cortisol and β-endorphin before, during and after separation (SEP) in seven goats. Blood samples were taken 30 and 5 minutes before separation, during separation at 10.30 h, and 5, 15, 30, 60 and 120 minutes after separation. ** = P ≤ 0.01 indicates significant difference compared to the first sample (-30 minutes).

There were no changes in heart rate and blood pressure at separation (Figure 4). The plasma concentration of cortisol increased temporarily in the second control sample (5 minutes before separation) (P ≤ 0.01) but decreased at separation and stayed low during 120 minutes (Figure 5). Plasma β-endorphin did not change during separation (Figure 5). In the first control sample, the concentration of oxytocin was 22 ± 2 pmol/l and the concentration of vasopressin was 1.0 ± 0.10 pmol/l, and did not differ significantly during the experiment.

**Figure 6:**
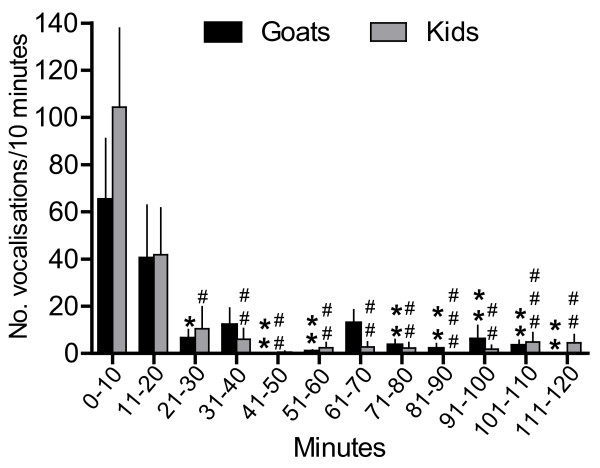
**Vocalisation in goats and kids after separation (Study 3)**. Means (± SEM) of number of vocalisations/10 minutes in seven goats and their seven kids after they were separated at time 0 minutes. * = P *≤ *0.05, ** = P *≤ *0.01 and *** = P *≤ *0.001 indicate significant differences compared to the first 10 minutes period (0-10 minutes) for the goats and **^# ^**= P *≤ *0.05, **^# # ^**P *≤ *0.01 and **^# # # ^**= P *≤ *0.001 indicate significant differences compared to the first 10 minutes period (0-10 minutes) for the kids.

During the first 10 minutes period after separation, both goats and kids vocalised intensively and vocalisation rate remained high during the subsequent 10 minutes period. During the remaining periods, vocalisation rate was lower in both goats and kids, except for 31-40 minutes and 61-70 minutes after separation in the goats (Figure 6). The kids tended to vocalise more than the goats during the first 10-minutes period (P = 0.08). After separation, the latency until lying down in the goats was 11 ± 3 minutes.

### Study 4, Heart rate, blood pressure and time spent lying down at nights

**Figure 7:**
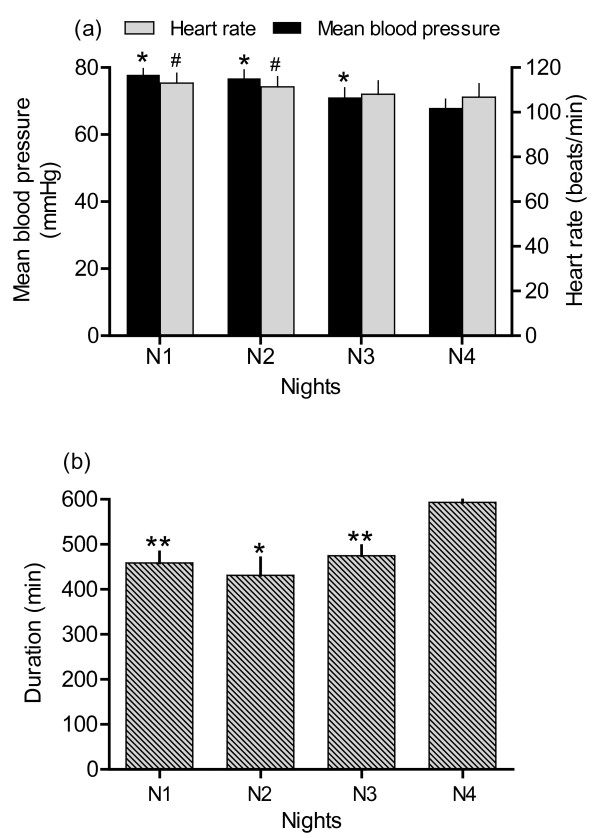
**Heart rate, blood pressure and time spent lying down at nights (Study 4)**. Means (± SEM) of heart rate and mean arterial blood pressure in seven goats (a) and duration of lying down (b) during the first night after parturition (N1), the second night after parturition (N2), the night after Study 2 (N3), and the night after Study 3 (N4). Differences between nights were calculated from mean values between 18.00 h and 06.00 h when the goats were left undisturbed. * = P ≤ 0.05 indicate significant difference compared to the night after Study 3 (N4) for blood pressure and ^#^= P ≤ 0.05 for heart rate (a). * = P ≤ 0.05 and ** = P ≤ 0.01 indicate significant difference compared to the night after Study 3 (N4) in duration of lying down (b).

The heart rate was significantly lower the night after Study 3, when goat and kid was separated, than in the first and second nights after parturition (P ≤ 0.05) (Figure 7a). Mean blood pressure decreased gradually and was lower the night after Study 3 than all earlier nights (P ≤ 0.05) (Figure 7a). The night after Study 3, the goat and kid separation, the goats spent more time lying down than all earlier nights (Figure 7b).

## Discussion

Suckling in goats was accompanied by increased cortisol and β-endorphin concentrations, but with no significant changes in heart rate and blood pressure. Permanent separation caused no changes in arterial blood pressure, heart rate, or plasma concentrations of cortisol and β-endorphin, although intensive vocalisation occurred during the first 20 minutes.

A small but not significant increase in blood pressure was observed at suckling, when the recordings were taken in undisturbed goats. When suckling was combined with blood sampling, there was more variation in both blood pressure and heart rate. Possibly, a more general arousal in the goats occurred during these days, *i.e *the activity in the animal facilities might have made the goats more alert, which affected the cardiovascular variables. No change in heart rate in response to suckling was observed. These results can be compared with an increase of about 6% in blood pressure and about 10% in heart rate, respectively, during hand milking [23]. In that experiment goats were lightly restrained and blood sampling took about 3 minutes compared with less than one minute in the unrestrained goats in the present experiment.

Both plasma cortisol and β-endorphin increased during suckling and in the sample taken 5 minutes after suckling, which supported that suckling activated the hypothalamic-pituitary-adrenal axis. Increased plasma cortisol concentrations have been found during and immediately after milking in suckled cows [26,27], in cows milked by hand or machine [28], in ewes during suckling or machine milking [29], and in goats during suckling and hand milking [23]. The consistent increase in plasma cortisol concentration during and after milking suggested the metabolic effects of cortisol was the reason for the increase, although a combination with some arousal cannot be excluded. Cortisol is released from the anterior pituitary upon stimulation by ACTH. The importance of ACTH and other anterior pituitary hormones for the development of the mammary gland and maintenance of milk secretion have been demonstrated already in 1963 in goats by Gale [30] and Gale and Larsson [31].

There was no elevation of either plasma oxytocin or vasopressin concentrations. This contrasted with the study by Olsson and Högberg [23] who determined elevated plasma oxytocin and vasopressin concentrations during suckling in goats. However, in that study, the kids suckled for about 3 minutes after they had been separated from their mothers for a couple of hours. In this study, kids suckled during a mean of 43 seconds and were together with their mothers the whole time. In the study by Olsson and Högberg [23], several blood samples were taken during one suckling. Hence, a short lasting change could have been missed in the single blood sample taken in this study. In addition, the goats had been lactating for 6-8 weeks in the previous study, compared to 3-5 days in this study, which may have influenced the differences between the studies. Goats have a large fraction of milk in the cistern [32], and according to McNeilly [33], oxytocin is not essential for milk removal during suckling in the goat. Suckling occurred frequently in the present study and was presumably often only an emptying of cisternal milk. In addition, increased β-endorphin levels, which was found in the present study, are accompanied by decreased oxytocin release in cows milked in unfamiliar surroundings [34,35].

Sudden and permanent separation did not alert the sympathetic nervous system as indicated by the unchanged heart rate and blood pressure. The lack of cortisol response was in accordance with earlier separation studies in goats [10], cows [36], and ewes [37]. Combined with the unchanged β-endorphin concentration, the results indicated that the separation procedure did not activate the hypothalamus-pituitary-adrenal axis.

One explanation for the lack of change in the physiological variables during and after separation could be that goats and kids, unlike lambs which are followers, have only contact for nursing the first days after parturition [7,8] and may be adapted to periods of separation, as suggested in calves [3]. Another explanation is that the distension of the udder and satiety of the kid influences the response to separation [38]. The behavioural response to separation is less pronounced in both goats and kids when the kids were allowed to suckle before separation than when suckling had been prevented 2 hours prior to separation [38]. In the present study, the goats and kids were together until the time of separation. Therefore, the goats were probably not upset due to a filled udder or calls from a hungry kid after separation. This was supported by the observation that vocalisation was intensive only during the first 20 minutes and subsided thereafter. It did not take long for the goats to lie down after separation, although there were some individual differences, as in cows separated from their calves [39]. The goats did not display behavioural or physiological signs of distress, even several hours after separation, (*i.e*. during the night after separation). The goats were milked in the afternoon and the kids fed, which meant they were not suffering from a distended udder or hunger.

Heart rate is higher during daytime than night time during both pregnancy and lactation, but with no diurnal variation in blood pressure [16]. In the present study, blood pressure and especially heart rate varied according to the activity level of the goats and events in their environment during daytime. At night, the goats stood more when they were together with their kids. This can partly be due to the goats standing up when the kids were suckling. In addition, goat mothers are alert and ready to aggressively protect their kids if needed [40]. This could explain why blood pressure and heart rate was higher during the nights before separation than after separation. In other studies[16,24], kids were separated immediately post partum and blood pressure did not change in the goats during the week after parturition, while the heart rate gradually decreased. This physiological response to adaptation from pregnancy to lactation could be part of the reason for the lowering of heart rate in the present study. Hence, the goats did not appear negatively affected by the separation, even though the goats and kids spent enough time together to develop mutual bonding. Both the physiological and behavioural effects were small after separation. However, it should be taken into consideration that separating bonded individuals means loss of pleasurable experiences, such as physical contact, grooming and nursing [1]. In addition, contact between mother and young for a longer period may be beneficial for the offspring, as is suggested for calves, where individuals separated seven days post partum coped better to a new environment than calves separated on the first day did [3].

## Conclusions

In conclusion, suckling elevated plasma cortisol and β-endorphin concentrations in the goats. The intensive vocalisation in the goats after separation, earlier suggested to indicate stress, was not accompanied by cardiovascular or endocrine responses.

## Competing interests

The authors declare that they have no competing interests.

## Authors' contributions

The study was designed by LWvW, EHS and LL. LWvW and EHS carried out the study. LWvW, EHS and LL analysed the data and AM performed the statistical analyses. LWvW and EHS drafted the manuscript and LL, KD and AM participated in the writing. All authors read, revised and approved the final manuscript.
